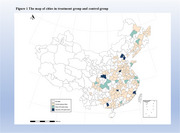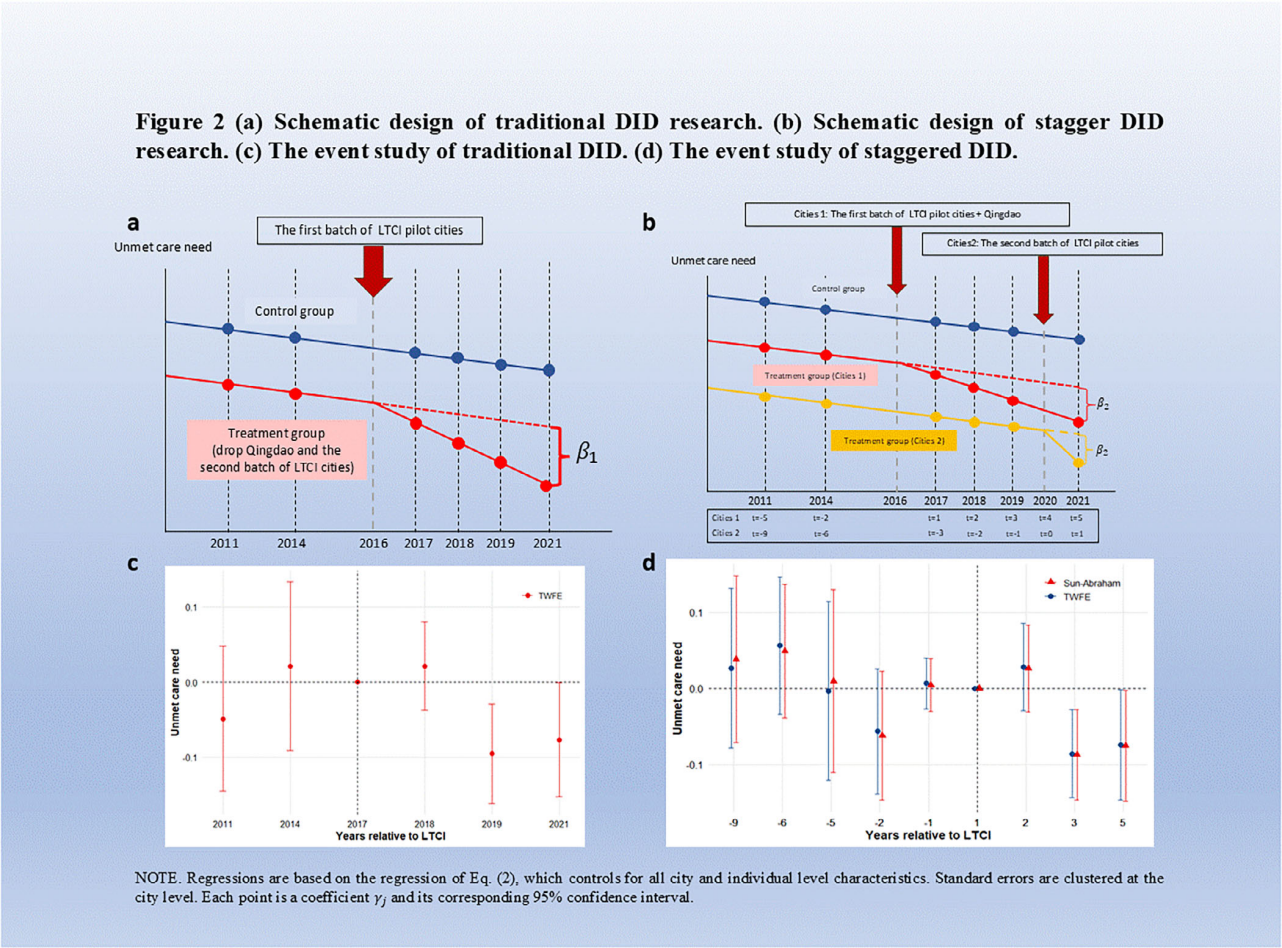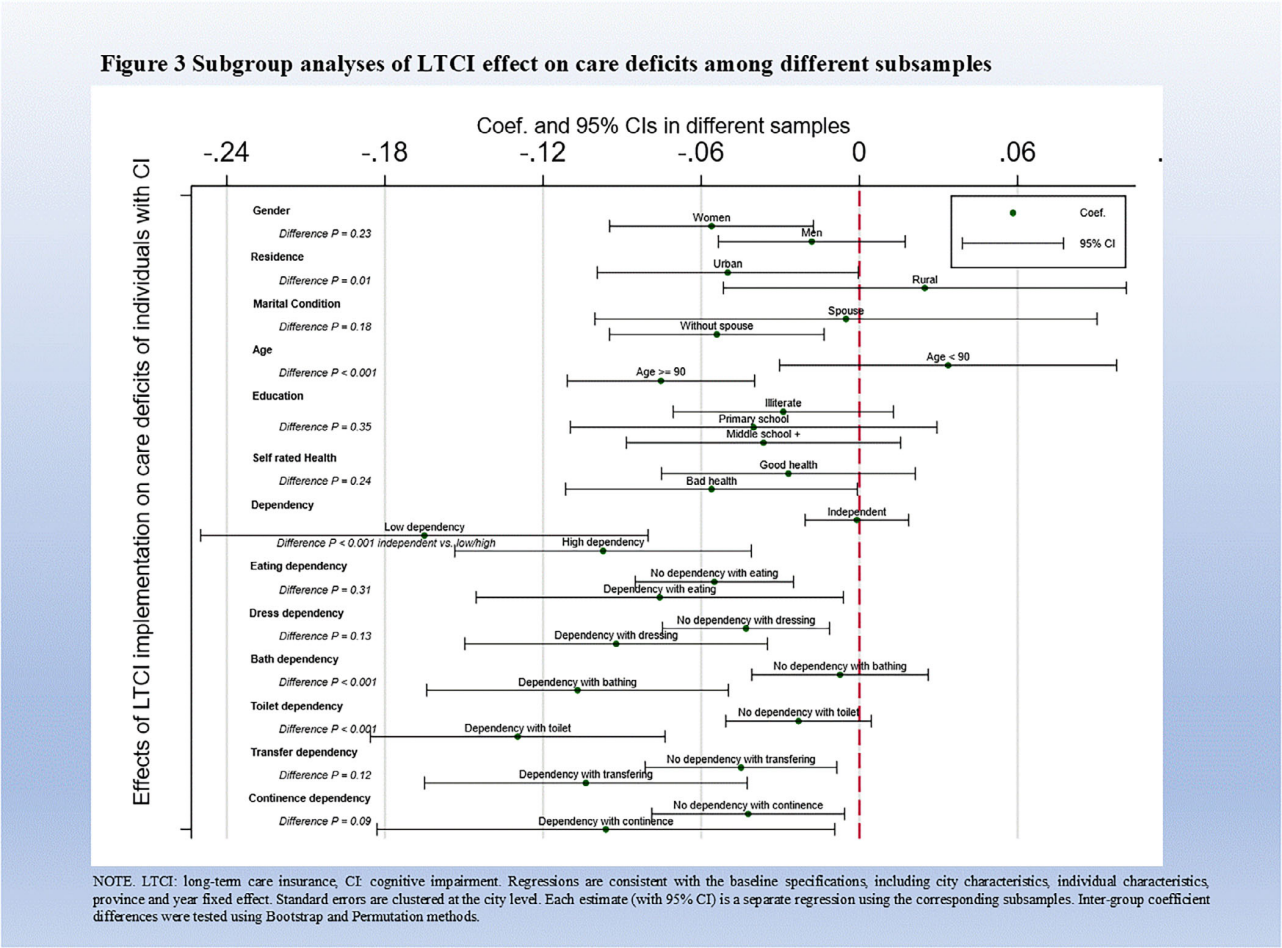# Does the long‐term care insurance policy meet the care needs of older individuals with cognitive impairment? a nationwide quasi‐experimental study

**DOI:** 10.1002/alz70858_101812

**Published:** 2025-12-25

**Authors:** Yuling Jiang, Wenjian Zhou, Wei Yang, Bo Hu, Yao Yao

**Affiliations:** ^1^ Peking University, Beijing, Beijing, China; ^2^ King's College London, London, London, United Kingdom; ^3^ London School of Economics and Political Science, London, London, United Kingdom

## Abstract

**Background:**

Unmet care needs and caregiving burdens for individuals with cognitive impairment (CI) are pressing global concerns, particularly in China. In 2016, China launched a long‐term care insurance (LTCI) program, yet its impact on unmet care needs for individuals with CI remains unclear.

**Method:**

This quasi‐experimental study leveraged data from the Chinese Longitudinal Healthy Longevity Survey (CLHLS; 2011, 2014, 2018, 2021), comparing outcomes before and after LTCI implementation. Participants in LTCI pilot cities were the intervention group, while those in non‐pilot areas were assigned as controls. Cognitive status was identified via Mini‐Mental State Examination scores, and unmet care needs were defined as the absence or inadequacy of needed care. A two‐way fixed‐effects difference‐in‐differences (TWFE‐DID) model was employed to estimate the impact of LTCI.

**Result:**

LTCI implementation reduced the risk of unmet care needs by 4.3% (95% CI: ‐0.083, ‐0.003; *p* = 0.036) and informal family caregiving time by 25.3% per week (95% CI: ‐0.503 to ‐0.003; *p* = 0.046). Benefits on unmet care needs were greater among urban residents (β: ‐0.050 vs 0.024; *p* = 0.01) and individuals aged ≥90 years (β: ‐0.075 vs 0.034; *p* < 0.001). The results remained steady within a set of robustness check.

**Conclusion:**

China's LTCI program significantly reduced unmet care needs and alleviated family caregiving burdens for older adults with CI, particularly among urban residents and those aged 90 years old and over. Expanding LTCI coverage to rural populations and younger individuals could enhance equity and broader benefits.